# Shedding Light on Skin Autoimmunity: More than Just Skin Deep

**DOI:** 10.3390/ijms241512077

**Published:** 2023-07-28

**Authors:** Philip Hei Li, Chak-sing Lau

**Affiliations:** Division of Rheumatology and Clinical Immunology, Department of Medicine, University of Hong Kong, Queen Mary Hospital, 102 Pokfulam Road, Pokfulam, Hong Kong

Our skin is the largest organ of the body and the foremost defensive barrier against the external environment. It is also regarded as an important immune organ, with resident immune cells of the skin responsible for immune surveillance and homeostasis. Therefore, the failure or breakdown of such critical processes plays an important role in the pathogenesis of many autoimmune disorders. These conditions, such as chronic spontaneous urticaria (CSU), cutaneous lupus erythematosus (CLE) and pemphigus, have a significant impact on the quality of life of affected individuals. Involvement of the mucosal surfaces, such as the conjunctivae, in autoimmune ocular surface diseases, may lead to severe sequalae such as visual impairment or even blindness. Despite significant advances in understanding the pathogenesis of these various disorders, there remains a need for more translational research to further advance the available therapeutic options. This Special Issue of the *International Journal of Molecular Sciences* features a range of papers demonstrating the dermatology and immunology research being carried out in the emerging field of skin autoimmunity.

One of the key themes in this Special Issue is the unravelling of the pathogenesis of various autoimmune skin disorders. Several papers in this Special Issue have focused on elucidating the molecular and cellular mechanisms underlying skin autoimmunity. For example, Amagai et al. [1] demonstrate that LL-37, an active form of cathelicidin, is generally dependent on scavenger receptors. Excess LL-37 is thought to enhance the local tissue inflammatory response and has been implicated in various autoimmune disorders. Therefore, inhibitors of scavenger receptors may potentially serve as new anti-inflammatory or immunosuppressive agents in the future. With a more specific focus on CSU, Gomułka and Mędrala [2] report that the vascular endothelial growth factor, platelet-activating factor, and eosinophil-derived neurotoxin were higher in patients with CSU compared with controls. These molecules may be directly involved in signaling cascades, chemotaxis, or cell degranulation, or they may reflect the “priming” of basophils or eosinophils as part of CSU pathogenesis. Although not correlating with disease severity, their findings may highlight a functional role of these cytokines in the disease’s pathogenesis. These studies not only deepen our understanding of the underlying biology of these conditions, but also suggest potential targets for future therapies.

Another important area of research highlighted in this Special Issue is the use of various models in the study of autoimmune skin conditions. In their comprehensive review, Lotti et al. [3] review the various in vitro, ex vivo and in vivo, as well as passive and active mouse models, that have been used in the study of pemphigus. Pemphigus is a very complex and heterogeneous disease, and these different disease models also allow for the evaluation of specific autoantibodies and the investigation of novel autoantigens. This knowledge could be harnessed to reproduce different pemphigus subgroups based on the involvement of different autoantigens.

This Special Issue also features several papers discussing novel therapeutic approaches for treating autoimmune mucocutaneous disorders. CLE is an autoimmune disorder, characterized by autoantibody production and immune cell recruitment. Soto et al. [4] review the pathogenesis and experimental treatments in various types of CLE in both animal models and clinical trials. Ling et al. [5] describe the potential role of Dendrobium, a traditional Chinese medicine used as both food and medicine, in alleviating dry eye disease in a rat model. Their study demonstrates that oral administration of Dendrobium extracts may enhance tear production in rats and has a protective effect on ocular surface damage. These new perspectives on the treatment of dry eye disease suggest the possible therapeutic role of Dendrobium in the future.

Overall, the unique papers in this Special Issue of the *International Journal of Molecular Sciences* provide valuable insights into the complex mechanisms governing autoimmune skin disorders and have highlighted the potential of novel therapeutic strategies. The field of skin autoimmunity shows considerable promise, and there is still much to be learned. As our understanding of skin autoimmunity continues to grow, we hope that this Special Issue serves as a catalyst for further research and innovation in this developing field of immunology and dermatology.

## Data Availability

Not applicable.
